# The Great American Biotic Interchange revisited: a new perspective from the stable isotope record of Argentine Pampas fossil mammals

**DOI:** 10.1038/s41598-020-58575-6

**Published:** 2020-01-31

**Authors:** Laura Domingo, Rodrigo L. Tomassini, Claudia I. Montalvo, Dánae Sanz-Pérez, María Teresa Alberdi

**Affiliations:** 1 Departamento de Geodinámica, Estratigrafía y Paleontología, Facultad Ciencias Geológicas, Universidad Complutense de Madrid, Madrid, 28040 Spain; 2 Earth and Planetary Sciences Department, University of California Santa Cruz, Santa Cruz, California, 95064 USA; 3 INGEOSUR, Universidad Nacional del Sur (UNS)-CONICET, Bahía Blanca, 8000 Argentina; 4 Facultad de Ciencias Exactas y Naturales, Universidad Nacional de La Pampa, Santa Rosa, 6300 Argentina; 5 Departamento de Paleobiología, Museo Nacional de Ciencias Naturales-CSIC, Madrid, 28006 Spain

**Keywords:** Palaeontology, Biogeochemistry, Evolution, Biogeochemistry, Ecology, Biogeochemistry, Palaeoecology, Stable isotope analysis, Biogeochemistry, Palaeoecology, Stable isotope analysis

## Abstract

This study aims at assessing resource and habitat use, niche occupation and trophic interactions from a stable isotope perspective on fossil mammals from the Argentine Pampas during the Great American Biotic Interchange (GABI). We present stable isotope data of more than 400 samples belonging to 10 mammalian orders and spanning a temporal range from ~9.5 Ma to ~12 ky. Rodents, notoungulates and pilosians record an increase in the consumption of C_4_ plants, whereas litopterns and cingulates show δ^13^C values that remain mostly within a C_3_-dominated diet. Our stable isotope data indicates that the expansion of C_4_ vegetation opened up new niche opportunities, probably alleviating resource competition among endemic taxa. Gomphothere, equid and camelid δ^13^C records show a broad variability pointing to consumption of C_3_ and mixed C_3_-C_4_ vegetation. This flexible dietary behavior may have facilitated the successful settlement of immigrant groups in South America. In the case of carnivorous taxa, Late Miocene pre-GABI endemic sparassodonts consumed prey from C_3_ environments, whereas immigrant carnivorans preferred prey from mixed C_3_-C_4_ areas. Our research contributes to the study of the GABI from a different perspective as stable isotope records permit to characterize, from a (semi)quantitative standpoint, ecological traits within extinct fauna.

## Introduction

Understanding the evolution of mammalian communities throughout the history of Earth, their resource and habitat use, niche occupation and trophic relationships play fundamental roles as these variables determine interaction among species, which ultimately trigger profound changes in community structure leading to modern faunal structure^1^.

The Late Cenozoic fossil record of South America provides a unique natural laboratory to investigate faunal response in the context of changing biotic and abiotic forces^2^. Recent works on the tectonism, paleoceanography, paleobiogeography and paleobiology of the Isthmus of Panama have refined our knowledge of its final formation, although not without controversy and sometimes opposing views about its tempo and mode^3^. Despite debates about tectonic *vs*. ecological barriers to intercontinental faunal exchange, most workers accept that South America was mostly isolated from other continents for more than 50 Ma, from the Late Paleocene until regular terrestrial faunal exchange began following the establishment of the Panama corridor^4,5^.

The Great American Biotic Interchange (GABI) occurred in pulses, reflecting intermittent connections due to sea level lowstands and glacial-interglacial climatic dynamics^4,6–8^. The first arrival of North American taxa into South America took place in the Late Miocene (Huayquerian) before the total establishment of the Panama corridor. Herald taxa were procyonids, cricetids and later on, tayassuids. Then, once the land bridge was completed by 3.1–2.7 Ma, the full GABI took place, with the entrance of other immigrant groups^8^. The magnitude of initial biotic interchange during GABI was roughly proportional to the diversity of each “donor” continent, but North American mammals radiated more successfully in South America^7,9^. While there is a good chronology of the GABI pulses, little is known about the resource and habitat use, niche occupation and trophic interactions in South America related to this event or the extent to which the arrival of northern mammals affected ecological attributes. In this sense, previous stable isotope studies on vertebrate taxa have greatly contributed to unveil these questions^10–17^.

Through the investigation of stable isotope data on a complete and updated record of Late Miocene to Late Pleistocene fossil mammals from Argentina, we aim at assessing whether (i) mammalian resource and habitat use and therefore, niche occupation changed throughout the GABI time span; and (ii) the successful radiation of northern immigrants in South America was related to dietary behavior and habitat use (in particular, niche breadth), which might have provided a competitive advantage relative to southern residents.

**Figure 1:**
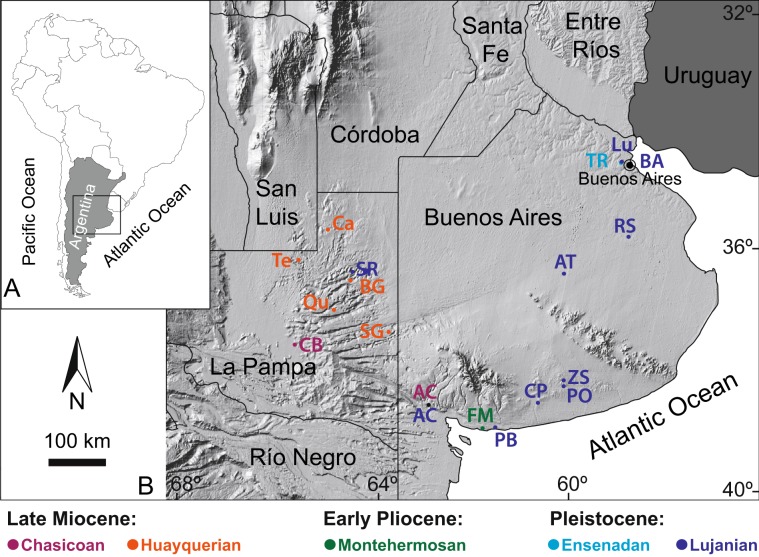
Geographical setting of the studied paleontological localities. (**A**) General map of South America. (**B**) Detailed map showing the situation of the Late Cenozoic fossil sites from Argentina (La Pampa and Buenos Aires provinces) selected in this study. CB: Cerro La Bota, AC: Arroyo Chasicó, Te: Telén, Qu: Quehué, SG: Salinas Grandes de Hidalgo, BG: Bajo Giuliani, Ca: Caleufú, FM: Farola Monte Hermoso, TR: Toscas del Río de La Plata, Lu: Luján, BA: Buenos Aires city area, RS: Río Salado, AT: Arroyo Tapalqué, PO: Paso Otero, CP: Cascada del Paleolama, PB: Playa del Barco, SR: Santa Rosa, ZS: Zanjón Seco. Samples from Arroyo Chasicó locality include fossils from Late Miocene (Chasicoan = C) and the Late Pleistocene (Lujanian = L) levels. Satellite images were freely downloaded from the Instituto Geográfico Nacional de Argentina. QGIS Desktop 3.2.3 with GRASAS 7.4.1 free software was used to assemble images (https://www.qgis.org/es/site/).

Selected fossiliferous localities situated in the Pampean region (La Pampa and Buenos Aires provinces), include faunal associations that represent different stages/ages^18,19^ (and references therein) spanning a temporal interval from pre-GABI times ~9.5 million years (Late Miocene, Chasicoan) to ~12,000 years (Late Pleistocene, Lujanian) (Fig. 1). In the present study, we analysed carbon and oxygen stable isotope compositions on fossil tooth enamel belonging to 8 mammalian orders and orthodentine belonging to xenarthran remains (Pilosa and Cingulata) from Pampean localities (Fig. 1, Supplementary Tables S1 and S2). Stable isotope analysis constitutes a proxy widely used to characterize paleoecological, paleoclimatic and paleoenvironmental variability (e.g.^13,20^; see Supplementary Text). Bioapatite δ^13^C values allows the characterization of the diet of extinct taxa, as well as the reconstruction of past habitat preferences, whereas bioapatite δ^18^O values record the δ^18^O value of body water reflecting changes in the isotopic composition of ingested water, which mainly varies with mean annual temperature and aridity^20^. By focusing on a specific region, we can overcome stable isotope biases due to particular hydrological, geographical, and/or vegetational conditions that may have existed among different geographical areas. Repeated stable isotope analyses on selected samples strongly support the reproducibility of the data (see Supplementary Text). Analytical methods follow well-established protocols for carbon and oxygen stable isotope analyses (see the Materials and methods section and Supplementary Text).

## Results and Discussion

### δ^13^C change through the GABI temporal interval

**Figure 2:**
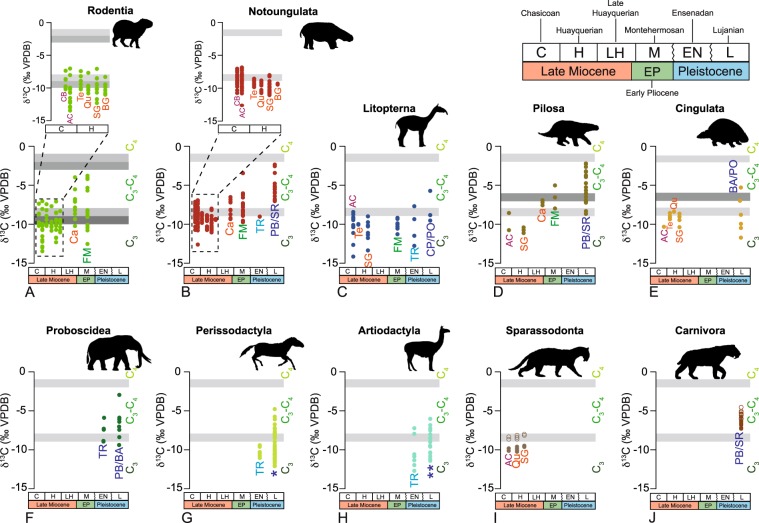
Bioapatite δ^13^C (‰, VPDB) values of 10 orders of herbivore and carnivore mammals from the La Pampa and Buenos Aires provinces between the Late Miocene (Chasicoan) and the Late Pleistocene (Lujanian). (**A**) Rodentia, (**B**) Notoungulata, (**C**) Litopterna, (**D**) Pilosa, (**E**) Cingulata, (**F**) Proboscidea, (**G**) Perissodactyla, (**H**) Artiodactyla, (**I**) Sparassodonta, (**J**) Carnivora. The grey bars represent the vegetation δ^13^C cut-off values between a C_3_-dominated diet, an intermediate C_3_-C_4_ diet, and a C_4_-dominated diet. The lightest grey denotes a ε*_diet-enamel_ of +14.1‰^21^, whereas the darkest one corresponds to a ε*_diet-enamel_ of +12.8‰ for rodents^22^, and ε*_diet-enamel_ of +15.6‰ for xenarthrans^23^. Sparassodonts and carnivorans show raw δ^13^C data (solid symbols) and δ^13^C data corrected for the trophic offset (open symbols), adding 1.3‰ to the raw values^24^. Locality abbreviations are the same as in Fig. 1. *Late Pleistocene (Lujanian) perissodactyl samples come from Luján, Río Salado, Arroyo Tapalqué, Cascada del Paleolama, Zanjón Seco, Paso Otero and Arroyo Chasicó localities (Supplementary Table S1). **Late Pleistocene (Lujanian) artiodactyl samples come from Playa del Barco, Santa Rosa, Cascada del Paleolama and Paso Otero localities (Supplementary Table S1). Zig-zag lines in the chronology tables denote non-represented ages: the Montehermosan is followed by the Chapadmalalan, the Marplatan, the Ensenadan, the Bonaerian, and the Lujanian.

Bioapatite δ^13^C values for Rodentia, Notoungulata, Litopterna, Pilosa, Cingulata, Proboscidea, Perissodactyla, Artiodactyla, Sparassodonta and Carnivora are shown in Fig. 2 and Supplementary Tables S1 and S2 As for the dietary-bioapatite δ^13^C enrichment (ε*_diet-enamel_), we use ε*_diet-enamel_ of +14.1‰^21^ for all orders (except for rodents and xenarthrans), +12.8‰ for rodents^22^, and +15.6‰ for xenarthrans^23^. Carnivorans´ trophic fractionation was also accounted for^24^ (see Supplementary Text).

The most noticeable feature is an overall increase of δ^13^C values for some of the endemic herbivore mammals (rodents, notoungulates, pilosians) recorded between the Late Miocene and the Pleistocene (Fig. 2). The increase in the bioapatite δ^13^C values of these taxa is intrinsically related to a shift in the use of plant resources from C_3_-dominated vegetation to mixed C_3_-C_4_ vegetation. The expansion of C_4_-dominated grasslands in Argentina has been recorded at ~8–7 Ma^11^. The rising of C_4_ plants at the Pampean region is framed within the timing of the “Edad de las Planicies Australes” (“Age of Southern Plains”), a period between 11 and 3 Ma characterized by an increase in aridity, a gradual decrease in temperatures and the establishment of vast grasslands after the retreat of the Paranean Sea^18^. The shift in our stable isotope data support the premise posed by MacFadden *et al*.^10^, who stated that after ~8 Ma, the Pampean ecosystem was characterized by a mix of C_3_ trees, shrubs and grasses, as well as C_4_ grasses. Unfortunately, there is not a good paleobotanical/palynological record in the Pampean deposits^2^, which makes the study of stable isotopes on vertebrate bioapatite of utmost interest in the understanding of vegetation evolution in the Pampean area during the late Cenozoic.

Representatives of the orders Rodentia and Notoungulata show relatively high bioapatite δ^13^C values at the Chasicoan (∼9.5 Ma, Late Miocene), including Arroyo Chasicó and Cerro La Bota^25^ localities, that remain close to the δ^13^C cut-off value between a C_3_-dominated diet and a C_3_-C_4_ diet (Fig. 2A,B). As the proportion of C_4_ plants within Pampean ecosystems may not have been significant until the latest Miocene, these high bioapatite δ^13^C values may be pointing to the consumption of C_3_ plants from xeric environments. However, we cannot fully discard the fact that C_4_ plants may have already found favorable conditions to thrive at this early age in the Pampean area as also suggested by Hynek *et al*.^12^ for the Northwest of Argentina. Rodentia, Notoungulata and Pilosa fed on a C_3_-C_4_ environment later in the Late Miocene/Early Pliocene Caleufú^26^ and Early Pliocene Farola Monte Hermoso^27^ localities (Fig. 2A,B,D). This trend towards an increase in the use of C_4_ resources is not as marked in Litopterna and Cingulata, which show δ^13^C values that remain mostly within a C_3_-dominated diet throughout the studied temporal interval (Fig. 2C,E). During the Pleistocene, notoungulates and pilosians show intermediate C_3_-C_4_ diets (Fig. 2B,D), whereas endemic litopterns and cingulates along with immigrant proboscideans, perissodactyls and artiodactyls show diets at the interplay between a C_3_-dominated diet and a mixed C_3_-C_4_ diet (Fig. 2C,E–H).

#### Rodentia

The dietary change recorded in rodent δ^13^C values is detected at the Late Miocene/Early Pliocene Caleufú and Early Pliocene Farola Monte Hermoso localities (Fig. 2A). This shift is statistically significant when compared to older localities from the Late Miocene (Arroyo Chasicó and Salinas Grandes de Hidalgo) (Supplementary Table S3). In our study, evaluated remains from Caleufú include Caviidae and Dinomyidae, whereas Farola Monte Hermoso is represented by Caviidae and Chinchillidae. All of them have a wide range of δ^13^C values that was not observed among these same families in older localities (Fig. 2A, Supplementary Table S2). Therefore, studied rodents were able to incorporate C_4_ resources to their previous C_3_-dominated diet as soon as C_4_ vegetation was available in the region. This is in agreement with Hynek *et al*.^12^, who observed a wide δ^13^C range on rodent tooth enamel in the Pliocene deposits from Northwestern Argentina, evidencing the consumption of C_4_ vegetation at this time.

#### Notoungulata

Chasicoan notoungulates from the Arroyo Chasicó (Late Miocene) locality are represented herein by different taxa of the families Toxodontidae, Hegetotheriidae, Mesotheriidae and Homalodotheriidae (Supplementary Table S2). Only the first three are represented in Late Miocene Cerro La Bota (Chasicoan), Telén, Quehué and Salinas Grandes de Hidalgo (Huayquerian). Chasicoan notoungulates show a wide δ^13^C range indicative of dietary flexibility from more forested-arboreal areas to open grasslands within a C_3_-continuum (Fig. 2B, Supplementary Table S2). Some of the Arroyo Chasicó and Cerro La Bota notoungulate δ^13^C values surpass the vegetation δ^13^C threshold value between a C_3_-dominated diet and a C_3_-C_4_ diet. It is likely that they may be recording a C_3_ diet within open xeric conditions as C_4_ plants were not still present in significant proportions at this time^11^, although this result may as well point to the existence of favorable climatic and environmental conditions for C_4_ photosynthesis at this early time as suggested by Hynek *et al*.^12^. Huayquerian notoungulate δ^13^C record starts with a decrease indicating a pure-C_3_ diet at this time period (Fig. 2B, Supplementary Table S2). Therefore, the shift in tooth enamel δ^13^C values represents a real change in resource use (not family-biased) for the faunas assigned to the Huayquerian. As observed in rodents, notoungulates record a significant shift in their δ^13^C values from the most modern Neogene faunas onwards (Fig. 2B, Supplementary Tables S2 and S3). In this context, the notoungulates from the Late Miocene/Early Pliocene Caleufú and Early Pliocene Farola Monte Hermoso localities, represented in our study by Toxodontidae, Hegetotheriidae and Mesotheriidae, depict a wide range of tooth enamel δ^13^C values around the boundary between C_3_-dominated and C_3_-C_4_ diets (Fig. 2B, Supplementary Table S2). Our results suggest that notoungulates changed their dietary preferences at the end of Miocene-beginning of the Pliocene, taking advantage of the expansion of C_4_ vegetation in the region, with hegetotherids and mesotherids likely developing a more flexible resource use and toxodontids adopting a more specialised intermediate C_3_-C_4_ diet. Toxodontids (*Toxodon*) from the Quaternary Late Pleistocene Playa del Barco and Santa Rosa sites retained this newly adopted dietary behavior becoming important dwellers of intermediate C_3_-C_4_ areas (Fig. 2B, Supplementary Tables S2 and S3). Our results agree well with previous stable isotope analyses on notoungulates from other fossil sites in South America^12,20,28^. These studies argued that the notoungulate dietary shift is pinpointed between 7 and 5.6 Ma and that this shift was independent from the notoungulate family, as also suggested by our results.

#### Litopterna

Litopterns from the Late Miocene Arroyo Chasicó (Chasicoan) and Salinas Grandes de Hidalgo (Huayquerian) localities show a C_3_-dominated diet, with a wide range of δ^13^C values indicative of a continuum from forested areas to more open conditions (Fig. 2C, Supplementary Table S2). These results agree well with the δ^13^C values from Salinas Grandes de Hidalgo provided by MacFadden *et al*.^10^. Litopterns from the Early Pliocene Farola Monte Hermoso locality, represented by Macraucheniidae and Proterotheriidae, show a narrower range of tooth enamel δ^13^C values, but still within a C_3_-dominated diet (Fig. 2C, Supplementary Table S2). Overall, our litoptern δ^13^C record does not track the expansion of C_4_ plants in the region, contrary to rodents and notoungulates. With respect to the Quaternary taxa, Macraucheniidae from the Early Pleistocene Toscas del Río de La Plata locality (Ensenadan) still had a C_3_-dominated diet, although with some values close to the C_3_-C_4_ transition, whereas Late Pleistocene (Lujanian) *Macrauchenia* from Arroyo Chasicó, Paso Otero and Cascada de Paleolama shows a slight shift towards more mixed C_3_-C_4_ diet, although the low number of samples from this time period precludes us from making a more solid statement (Fig. 2C, Supplementary Table S2). Our results are supported by previous studies from the Quaternary localities of Camet Norte (Argentina)^15^ and Tarija (Bolivia)^29^.

#### Pilosa and Cingulata

A diagenesis test was performed to check whether the analysed xenarthran orthodentine retained the original isotopic signal (Supplementary Text, Supplementary Table S4). Bocherens *et al*.^16^ measured the difference between collagen and bioapatite δ^13^C values within a same specimen and suggested that glyptodonts (Cingulata) and ground sloths (Pilosa) would have been herbivorous. Although in our record Xenarthra data points are scattered and scarce, it is notable the shift observed in the pilosian δ^13^C values from a C_3_-pure diet at the Late Miocene to a C_4_-based diet at the Pliocene and Pleistocene (Fig. 2D). Nothrotheriidae from the Late Miocene Arroyo Chasicó (Chasicoan) and Mylodontidae from Salinas Grandes de Hidalgo (Huayquerian) localities depict a δ^13^C value indicative of a diet based on C_3_ items. Later on, Mylodontidae from the Late Miocene/Early Pliocene Caleufú and Early Pliocene Farola Monte Hermoso localities record higher δ^13^C values pointing to a shift towards the consumption of C_3_-C_4_ vegetation from more open spaces, tracking the expansion of C_4_ plants in the region (Fig. 2D, Supplementary Table S2). Finally, pilosians from the Late Pleistocene Playa del Barco and Santa Rosa localities, represented by Mylodontidae and Megatheriidae, show a marked dietary change, with an overall consumption of C_3_-C_4_ resources (Fig. 2D, Supplementary Table S2). Cingulata orthodentine δ^13^C record (Fig. 2E, Supplementary Table S2) does not show such a marked shift through time: pampatheriids from the Late Miocene Arroyo Chasicó (Chasicoan) and glyptodontids and dasypodids from Telén, Quehué and Salinas Grandes de Hidalgo (Huayquerian) localities depict δ^13^C values indicative of a diet based on C_3_ items, whereas glyptodontids from localities from the Late Pleistocene Buenos Aires city area show a wide range of δ^13^C values, with most of individuals consuming C_3_-dominated resources and others incorporating mixed C_3_-C_4_ resources (Fig. 2E, Supplementary Table S2). Our Quaternary xenarthran results are in line with those reported by Bocherens *et al*.^15^, whose collagen δ^13^C analyses on Late Pleistocene cingulates and pilosians from the Buenos Aires province point to a greater consumption of C_4_ resources in the latter.

#### Proboscideans

Gomphotheriidae (*Stegomastodon*) from the Early Pleistocene Toscas del Río de La Plata and Late Pleistocene Playa del Barco and Buenos Aires city area localities shows wide ranges of δ^13^C values pointing to a flexible diet, which would have included pure-C_3_ and mixed C_3_-C_4_ plant resources (Fig. 2F, Supplementary Table S2).

#### Perissodactyla

Equids from the Early Pleistocene Toscas del Río de La Plata locality have δ^13^C values indicative of a C_3_-dominated diet. On the other hand, *Equus* and *Hippidion* from different Late Pleistocene (Lujanian) sites show a broad variability of δ^13^C values pointing to consumption of C_3_ and mixed C_3_-C_4_ vegetation (Fig. 2G, Supplementary Table S2), similarly to what is observed in the case of gomphotherids.

#### Artiodactyla

Artiodactyls from the Early Pleistocene Toscas del Río de La Plata locality are represented by Camelidae. *Lama* sp. records significantly lower δ^13^C values indicative of a browsing diet in a more wooded area, when compared to other camelids, assigned to?*Palaeolama* sp., with higher δ^13^C values pointing to a more intermediate diet (t = −1.361, p = 0.042) (Fig. 2H, Supplementary Table S2). At the Late Pleistocene Playa del Barco site, artiodactyls are represented by the cervid *Morenelaphus*, which shows the lowest δ^13^C values among our artiodactyl record (Supplementary Table S2), pointing to a browsing C_3_-diet and occupation of wooded areas. At the Late Pleistocene Santa Rosa locality, the camelids *Lama* and *Hemiauchenia* seem to have partitioned resources to some extent, with the former showing significantly lower δ^13^C values and the latter recording higher δ^13^C values (t = −3.492, p = 0.025) (Supplementary Table S2).

Dietary flexibility evidenced by northern immigrants (proboscideans, perissodactyls and artiodactyls) as shown by our δ^13^C values and other isotopic studies from North and South America^13,15,30–33^, and by extension, their plasticity in niche occupation, is in agreement with the habitat theory proposed by Vrba^34^ according to which generalists and open biome specialists from North America experienced a successful radiation throughout South America^34,35^.

#### Sparassodonta

Endemic sparassodonts are represented by the carnivorous and scansorial borhyaenoid *Lycopsis* from the Late Miocene Arroyo Chasicó site, and the hypercarnivorous thylacosmilid *Thylacosmilus* from the Late Miocene Quehué and Salinas Grandes de Hidalgo localities. Sparassodont δ^13^C record reveals a consumption of prey from C_3_ open areas (Fig. 2I, Supplementary Table S2). We used MixSIAR mixing model to estimate the proportions of source (prey) contributions to a consumer (predator)^36^ (see the Materials and methods section and the Supplementary Text). Late Miocene sparassodonts preferentially preyed on notoungulates (Supplementary Table S5).

#### Carnivora

Immigrant Carnivora from the Late Pleistocene Playa del Barco and Santa Rosa sites are represented by the hyper-carnivorous felid *Smilodon*, whose δ^13^C values point to the ingestion of prey species from mixed C_3_-C_4_ areas (Fig. 2J, Supplementary Table S2). MixSIAR results indicate that the analysed specimens from Playa del Barco preferentially preyed on notoungulates, whereas analysed specimens from Santa Rosa preferentially preyed on perissodactyls (Supplementary Table S5). In our study, *Smilodon* potential prey from mixed C_3_-C_4_ areas point to an intermediate situation between the ones provided by Cotte *et al*.^37^ and Bocherens *et al*.^15^. Cotte *et al*.^37^ carried out δ^13^C analyses on Late Pleistocene *Smilodon* tooth enamel from the Buenos Aires province and suggested that it mainly preyed on species from wet areas with C_3_ vegetation. Bocherens *et al*.^15^ performed δ^13^C analyses on Late Pleistocene *Smilodon* collagen from the same province, but their results point to *Smilodon*’s consumption of prey from open landscapes. This disparity may be indicative of its ability to hunt on a wide range of habitats and species.

Differences between Sparassodonta and Carnivora δ^13^C values are statistically significant (t = −17.746, p < 0.001) evidencing a different resource use and indirectly mirroring the expansion of C_4_ vegetation.

### Niche occupation through the GABI

**Figure 3:**
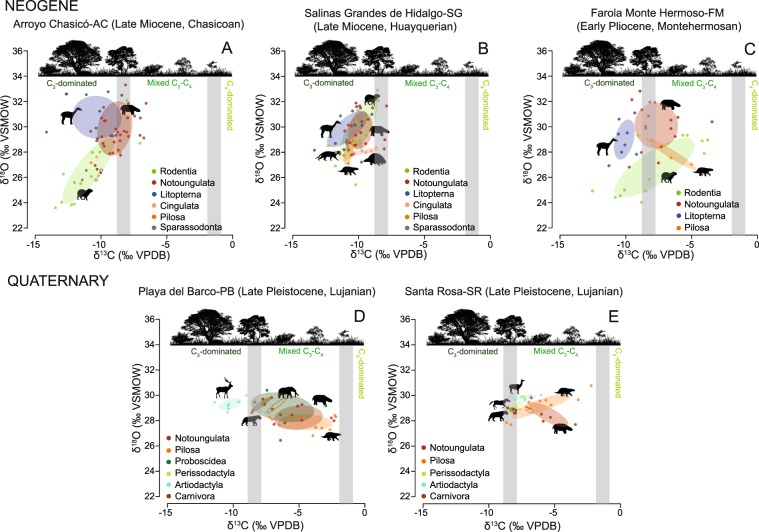
Standard ellipse areas (SEAs) generated by SIBER representing isotopic niches for selected Neogene and Quaternary localities. (**A**) Arroyo Chasicó, (**B**) Salinas Grandes de Hidalgo, (**C**) Farola Monte Hermoso, (**D**) Playa del Barco, and (**E**) Santa Rosa. The grey bars represent the transition between a C_3_-dominated diet, an intermediate C_3_-C_4_ diet and a C_4_-dominated diet. Sparassodonta and Carnivora ellipses show raw δ^13^C data (solid ellipse) and δ^13^C data corrected for the trophic offset (open ellipse), adding 1.3‰ to the raw values^24^.

Isotopic niches were assessed by using a Bayesian approach described in the Materials and methods section and the Supplementary Text. In the Pre-GABI Late Miocene Arroyo Chasicó locality, rodents, notoungulates and litopterns show some degree of ellipse (niche) overlap (Fig. 3A, Supplementary Text). Overall, taxa ingested vegetation from C_3_ areas, although notoungulates seems to have significantly incorporated C_3_ plants from more open spaces, whereas rodents and litopterns consumed C_3_ vegetation from more wooded areas (Supplementary Table S6). δ^18^O are driven by particular water use, ecologies and ecosystem preferences among different taxa and therefore, we consider this isotopic system in the assessment of habitat use, which along with the evaluation of resource use can help us depict niche occupation and width. In this sense, fitted loess δ^18^O curves along time depicted in Fig. S1 show different patterns for each order probably reflecting particular water use, rather than recording global climatic trends. At Arroyo Chasicó, rodents show significantly lower δ^18^O values (Fig. 3A, Supplementary Table S6) when compared to notoungulates and litopterns, which may point to ingestion of water (either via drinking or food) subject to a lower degree of evaporation. Rodents have narrower home ranges when compared to large mammals, so they could be tracking more local conditions, whereas notoungulates and litopterns would provide a more integrated signal of their broader home ranges^38^. In this sense, notoungulates and liptoterns do not show significantly different δ^18^O values (Fig. 3A, Supplementary Table S6), which point to ingestion of water subject to similar hydrological conditions. The δ^13^C-δ^18^O ellipses could not be retrieved for xenarthrans and sparassodonts since they have fewer than three datapoints, although most of them fall within a C_3_-dominated area (sparassodonts would have used prey from these areas). They show overall low δ^18^O values indicative of water ingestion with a lower degree of evaporation (Fig. 3A).

At the Late Miocene Salinas Grandes de Hidalgo locality, which evidences the first pulse of the GABI through the presence of the procyonid *Cyonasua* (not sampled in this study), there is a more intense ellipse (niche) overlap among the analyzed taxa (Fig. 3B). As observed in the case of the Arroyo Chasicó locality, all taxa consumed C_3_-dominated vegetation (or preyed on taxa with a pure C_3_ diet in the case of sparassodonts), but their δ^13^C values are more condensed (Supplementary Table S6) and reflect their coexistence in a C_3_ woodland to open C_3_ grassland (Fig. 3B). Also δ^18^O values show a more intense overlap (Supplementary Table S6), pointing to ingestion of water subject to similar hydrological conditions. The more intense ellipse (niche) overlap and the contraction of ellipse (niche) breadth observed at this locality when compared to the Arroyo Chasicó locality may be indicative of tougher conditions related to increasing aridity reported previously for the end of the Miocene^26,39^.

During the latest Miocene and Early Pliocene, the expansion of C_4_ plants at the Pampean area occurred^10,12^. If at this time some of the endemic herbivore taxa were able to incorporate C_4_ vegetation, this may have eased the competition among fauna and favored their sympatry. This is illustrated by the significant change in the δ^13^C values of rodents, notoungulates and pilosians at the Late Miocene/Early Pliocene Caleufú and Early Pliocene Farola Monte Hermoso localities (Fig. 2A,B,D). Farola Monte Hermoso rodents, notoungulates and pilosians retrieved ellipses that evidence a more intense use of open C_3_ and/or C_4_ vegetation when compared with previous times (Fig. 3C). Rodents show the widest range of δ^13^C values (Fig. 3C) pointing to the consumption of a broad array of resources that include both C_3_-dominated and mixed C_3_-C_4_ vegetation. Rodents also show significantly different δ^18^O values when compared to notoungulates and litopterns (Supplementary Table S6), pointing to ingestion of water subject to different hydrological conditions (less intense evaporation) probably related to more local conditions and the ecology of some groups (e.g., fossil capybaras-*Phugatherium*, Hydrochoeriinae, Caviidae, -are recorded associated to water bodies). Notoungulates and pilosians show high δ^13^C values indicative of the use of C_3_ and C_4_ plants from more open spaces when compared to litopterns (Fig. 3C; Supplementary Table S6). It is noticeable the fact that this relationship between notoungulates and litopterns is maintained since the Chasicoan, something that has been supported by other lines of evidence, such as dental and body morphology^19^. Overlapping δ^18^O values among notoungulates, litopterns and pilosians point to water use from sources subject to similar hydrological regime (Fig. 3C; Supplementary Table S6). Another interesting observation that is consistent at the Chasicoan, Huayquerian and Montehermosan (Fig. 3A–C) lies in the fact that rodent δ^13^C and δ^18^O values show a positive covariance. Lower resource δ^13^C values correspond to lower (drinking or food) ingested water δ^18^O and *vice versa* pointing to more closed or more open areas, respectively. This relationship is not observed in the case of larger taxa supporting the idea that rodents may be better recorders of ecosystem variability at a more local level.

North American herald taxa such as cricetids^26^ and procyonids^40^ are recorded in the Pampean region since the Late Miocene/Early Pliocene (Huayquerian). Then, the massive entrance of northern fauna took place once the Panama Isthmus was fully established. Therefore, the shift in vegetation use by endemic fauna evidenced by our isotopic data at the Caleufú (late Huayquerian) and Farola Monte Hermoso (Montehermosan) sites was not a consequence of pressure caused by new arrivals.

Global records point to optimal climatic conditions at this interval, when reorganized ocean circulation, perhaps associated with initial restriction of circulation between the Pacific and Atlantic, contributed to the Pliocene Warm Period between ~4.7 and 3.1 Ma^41^. Based on the faunal assemblage, warm conditions, with open xerophytic woodlands and grasslands, similar to today´s Chacoan phytogeographic province, but probably with greater humidity, were proposed for Farola Monte Hermoso locality. A global cooling coincident with expansion of ice in Antarctica happened at ~3.0 and 2.7 Ma, marking the beginning of the Quaternary^42^. The main GABI events are contemporary to this climatic deterioration, which accompanied by more arid conditions favored the development of open areas in South America and the establishment of a savanna corridor between North and South America facilitating the colonization by generalists and open biome specialists from North America^34,35^. South American endemic fauna experienced a large number of extinctions at the Late Pliocene (Sanandresian) and Early Pleistocene (Ensenadan)^18^. Although initial studies pointed to the arrival of northern immigrants as the trigger of this extinction events^43^, more recent research suggests that the entrance into the Pampean region was gradual and that the biomass represented by these new taxa was insignificant at this moment and therefore, the global cooling is proposed as the main triggering factor of the extinction^18,44,45^. The interaction with northern immigrants became more important since the Early Pleistocene (Ensenadan) and Middle Pleistocene (Bonaerian) and it was particularly keen during the Late Pleistocene (Lujanian). We lack isotopic data from the Bonaerian fauna, but our record of the Ensenadan and Lujanian endemic taxa, such as notoungulates and pilosians, shows a modification in their vegetation ingestion towards a more generalist intermediate C_3_-C_4_ diet (Figs. 2 and 3).

At the Late Pleistocene Playa del Barco locality, the faunal configuration is mainly characterized by the presence of large and megamammals. The analyzed taxa show a marked change in the resource use, with the full incorporation of mixed C_3_-C_4_ vegetation typical of grasslands at the Pampean area (Fig. 3D). Notoungulates, pilosians and perissodactyls almost exclusively consumed C_3_-C_4_, whereas proboscideans also incorporated plants from C_3_-dominated open areas (Fig. 3D, Supplementary Table S6). Felids (*Smilodon*) preyed on taxa that consumed mixed C_3_-C_4_ vegetation with mixing models pointing to notoungulates as their possible preferred prey (Fig. 3D, Supplementary Tables S5 and S6). Artiodactyla, exclusively represented by the cervid *Morenelaphus*, is the only order that shows a clear resource partitioning. Their significantly lower δ^13^C values evidences the consumption of C_3_ plants from more wooded spaces (Fig. 3D, Supplementary Table S6). Their higher δ^18^O values (Fig. 3D, Supplementary Table S6) may be related to either ingestion of drinking water or plant water subject to a higher degree of evaporation.

At the Late Pleistocene Santa Rosa locality, also large and megamammals are represented (Fig. 3E). Notoungulates and pilosians show similar dietary preferences (mixed C_3_-C_4_ vegetation) to that depicted at Playa del Barco. Artiodactyls are represented by camelids, whereas perissodactyls are represented by equids, both showing δ^13^C values indicative of a C_3_ diet from open areas and an intermediate C_3_-C_4_ diet (Fig. 3E). Similarly to that observed at Playa del Barco, felids (*Smilodon*) preyed on individuals that incorporated mixed C_3_-C_4_ vegetation, although the mixing model indicates that representatives of this locality probably preyed on perissodactyls (Fig. 3E, Supplementary Table S5). Santa Rosa taxa show overlapping and non-significantly different δ^18^O values (Fig. 3E, Supplementary Table S6), similar to those shown at Playa del Barco pointing to a similar hydrological regime.

Northern newcomers such as probocisdeans, perissodactyls and camelids had a flexible dietary behavior (from pure C_3_ to mixed C_3_-C_4_ diet, Fig. 2), which may have facilitated their successful radiation in South America as supported by their abundant fossil record in the subcontinent (see 13). The case of cervids is noticeable since they showed a more restricted diet based on plant resources from C_3_-dominated areas (Figs. 2 and 3D, Supplementary Table S2). Cervids may have successfully radiated into South America by taking advantage of resources from wooded areas within a savanna habitat. According to Cione *et al*.^18^, although the mammalian taxonomic diversity at the Late Pleistocene was high, the number of individuals per species and the total biomass were not probably elevated as most of the South American glacial ecosystems may not have been very productive. Following this statement, Playa del Barco and Santa Rosa localities may have represented low-productive ecosystems and therefore, resource partitioning would have been key to support endemic and non-endemic large mammalian coexistence. This seems to be the case for both localities, where notoungulates and xenarthrans seem to have relied more on mixed C_3_-C_4_ vegetation, gomphotheres, equids and camelids fed on C_3_ plants from open areas and intermediate C_3_-C_4_ vegetation, and cervids consumed C_3_ plants from wooded areas (Fig. 3D,E).

## Conclusions

The Argentine Pampas (Buenos Aires and La Pampa provinces) have proven to be one of the best research areas to study the GABI from the perspective of the South American fossil record on account of its elevated number of Late Cenozoic fossil sites with diverse and abundant vertebrate faunal remains. For this study, we carried out biopatite stable isotope analyses (δ^13^C and δ^18^O) on a significant number of Pampean fossil mammalian remains belonging to 10 orders and spanning a temporal range from ~9.5 million years (Late Miocene, Chasicoan) to ~12,000 years (Late Pleistocene, Lujanian). Rodents, notoungulates and pilosians track the expansion of C_4_ plants in the Pampean area during the latest Miocene/earliest Pliocene (Huayquerian/Montehermosan), whereas litopterns and cingulates kept mostly a C_3_-dominated diet. North American taxa including gomphotheres, equids and camelids record a large variability in their bioapatite δ^13^C pointing to consumption of C_3_ and mixed C_3_-C_4_ vegetation facilitating their successful settlement in the Pampean region. Cervids kept a C_3_-dominated diet, but were still able to thrive in their expansion into the South. In the case of carnivorous taxa, Late Miocene pre-GABI endemic sparassodonts preferentially preyed on species from open C_3_ environments, whereas immigrant carnivorans preferred prey from intermediate C_3_-C_4_ areas. Some of the selected taxa have never been explored from a stable isotope standpoint. Therefore, this study provides invaluable paleoecological and paleoenvironmental information from an isotopic perspective on late Cenozoic endemic and immigrant taxa implied in the GABI.

## Materials and Methods

For this study, we analysed the carbon and oxygen isotope composition on the carbonate fraction of 338 mammalian bioapatite (tooth enamel and orthodentine) samples spanning a temporal interval from the Late Miocene (Chasicoan) to the Late Pleistocene (Lujanian) of the Argentine Pampas (Buenos Aires and La Pampa provinces) (Fig. 1, Supplementary Tables S1 and S2). Additionally, 72 bioapatite samples belonging to different Pleistocene taxa from localities situated at the Buenos Aires province were included in the database from a previous study (see 13).

A rotary drill with a diamond-tipped dental burr was used to recover enamel and orthodentine from an area of the tooth as large as possible to avoid seasonal bias in the time of mineralization. The carbon and oxygen isotope results are reported in the δ-notation δ^H^X_sample_ = [(R_sample_ − R_standard_)/R_standard_]x1000, where X is the element, H is the mass of the rare, heavy isotope, and R = ^13^C/^12^C or ^18^O/^16^O. Vienna Pee Dee Belemnite (VPDB) is the standard for δ^13^C values, whereas Vienna Standard Mean Ocean Water (VSMOW) is the standard for δ^18^O values. Sample chemical treatment followed the one described in Domingo *et al*.^46^ for carbonate in bioapatite. Analyses were conducted at the Stable Isotope Laboratory of the University of California Santa Cruz using a ThermoScientific MAT253 dual inlet isotope ratio mass spectrometer coupled to a ThermoScientific Kiel IV carbonate device (see Supplementary Text for more details).

For diagenesis control, we measured the oxygen isotope composition of both the carbonate and phosphate fractions of 322 bioapatite samples (see Supplementary Text and Supplementary Tables S2 and S4). Sample chemical treatment followed the one described in Domingo *et al*.^46^ for phosphate in bioapatite. δ^18^O_PO4_ values were measured at the Stable Isotope Laboratory of the University of California Santa Cruz using a Thermo Finnigan Delta plus XP isotope ratio mass spectrometer coupled via continuous flow to a high temperature conversion elemental analyzer (TCEA) (see Supplementary Text for more details).

We used R software version 3.1.1. (http://www.r-project.org/)^47^ for statistical analysis (Supplementary Tables S3 and S6) and graphical representation of the isotopic results.

We used MixSIAR mixing model^36^ to estimate the proportions of source (prey) contributions to a consumer (predator) (Supplementary Table S5). Mixing models find the combinations of prey proportions that are mathematically feasible solutions that would explain consumer isotope values. With the aim of not compromising the discriminatory power of the mixing model due to a large number of sources, we kept the source number below 6. MixSIAR results (posterior probabilities of dietary proportions) are reported as the median and 95% Bayesian credible intervals of the likely contribution of each prey taxon to the tissue composition of the predators. MixSIAR uses a Markov Chain Monte Carlo (MCMC) model-fitting algorithm. We evaluated isotopic niches using SIBER (Stable Isotope Bayesian Ellipses in R^48^). To assess isotopic niche by means of SIBER it is necessary to use at least two isotopic systems (in our case, δ^13^C and δ^18^O), which permit to delimitate a 2D δ-space. SIBER also needs to be fed with at least three data point per taxon to retrieve ellipses. SIBER uses a Markov Chain Monte Carlo (MCMC) model-fitting algorithm to construct a standard ellipse area (SEA) that best fits each set of δ^13^C and δ^18^O from different members within a (paleo)community (i.e., different taxa). We evaluated the isotopic niche in those localities with a good representation of taxa (three or more orders) and where each order has a good sampling record to retrieve ellipses (three or more samples per order).

## Supplementary information

Supplementary Information.

Supplementary Information 2.

Supplementary Information 3.

Supplementary Information 4.

Supplementary Information 5.

Supplementary Information 6.

Supplementary Information 7.

Supplementary Information 8.

## Data Availability

All data generated or analysed during this study are included in this published article and in the Supplementary Information files.

## References

[1] Ezard THG, Purvis A (2016). Environmental changes define ecological limits to species richness and reveal the mode of macroevolutionary competition. Ecol. Lett..

[2] Ortiz-Jaureguizar E, Cladera GA (2006). Paleoenvironmental evolution of southern South America during the Cenozoic. J. Arid. Envir.

[3] O’Dea A (2016). Formation of the Isthmus of Panama. Sci. Adv..

[4] Webb, S. D. Late Cenozoic mammal dispersals between the Americas in *The* Great *American Biotic Interchange* (eds. Stehli, F. G., Webb, S. D.) 357–386 (Plenum Press, New York, 1985).

[5] Flynn JJ (2005). Geochronology of Hemphillian-Blancan aged strata, Guanajuato, Mexico, and implications for timing of the Great American Biotic Interchange. J. Geol..

[6] Simpson GG (1950). History of the fauna of Latin America. Am. Sci..

[7] Webb SD (2006). The Great American Biotic Interchange: patterns and processes. Ann. Mo. Bot. Gard..

[8] Woodburne MO (2010). The Great American Biotic Interchange: dispersals, tectonics, climate, sea level and holding pens. J. Mammal. Evol..

[9] Webb, S. D., Marshall, L. G. Historical biogeography of recent South American land mammals in *Evolution of Neotropical Mammals* (eds. Mares, M. A., Genoways, H. H.) 39–54 (vol. 6. Pymatuning Laboratory of Ecology, Univ. Pittsburgh, 1982).

[10] MacFadden BJ, Cerling TE, Prado JL (1996). Cenozoic terrestrial ecosystem evolution in Argentina: evidence from carbon isotopes of fossil mammal teeth. Palaios.

[11] Cerling TE (1997). Global vegetation change through the Miocene/Pliocene boundary. Nat..

[12] Hynek SA (2012). Small mammal carbon isotope ecology across the Miocene–Pliocene boundary, northwestern Argentina. Earth Planet. Sc. Lett..

[13] Domingo L, Prado JL, Alberdi MT (2012). The effect of paleoecology and paleobiogeography on stable isotopes of Quaternary mammals from South America. Quaternary Sci. Rev..

[14] Prevosti FJ, Martin FM (2013). Paleoecology of the mammalian predator guild of Southern Patagonia during the latest Pleistocene: Ecomorphology, stable isotopes, and taphonomy. Quat. Int..

[15] Bocherens H (2016). Paleobiology of sabretooth cat *Smilodon populator* in the Pampean Region (Buenos Aires Province, Argentina) around the Last Glacial Maximum: Insights from carbon and nitrogen stable isotopes in bone collagen. Palaeogeogr. Palaeocl Palaeoecol..

[16] Bocherens H (2017). Isotopic insight on paleodiet of extinct Pleistocene megafaunal Xenarthrans from Argentina. Gondwana Res..

[17] Cotte, M., Soibelzon, L., Prevosti, F. J., Vizcaino, S. F., Bocherens, H. Insights into the palaeoecology of Southern South America during the Neogene using the stable isotope analysis of mammalian tooth enamel and dentine carbonate. *SVP 76th Annual Meeting. Abstract volume* 121 (2016).

[18] Cione, A. L., Gasparini, G. M., Soibelzon, E., Soibelzon, L.H., Tonni, E.P. *The Great American Biotic Interchange. A South American perspective*. Springer briefs in Earth Sciences, 97 (2015).

[19] Croft, D. A. Horned armadillos and rafting monkeys in *Life of the past* (ed. Farlow, J. O.) 304 (Indiana University Press, 2017).

[20] Koch, P. L. Isotopic study of the biology of modern and fossil vertebrates in *Stable Isotopes in Ecology and Environmental Science* (eds. Michener, R., Lajtha, K.) 99–154 (2nd ed. Blackwell Publishing, Boston, 2007).

[21] Cerling TE, Harris JM (1999). Carbon isotope fractionation between diet and bioapatite in ungulate mammals and implications for ecological and paleoecological studies. Oecologia.

[22] Passey BH (2005). Carbon isotope fractionation between diet, breath CO_2_, and bioapatite in different mammals. J. Archaeol. Sci..

[23] Tejada-Lara JV (2018). Body mass predicts isotope enrichment in herbivorous mammals. Proc. R. Soc. B.

[24] Clementz MT, Fox-Dobbs K, Wheatley PV, Koch PL, Doak DF (2009). Revisiting old bones: coupled carbon isotope analysis of bioapatite and collagen as an ecological and palaeoecological tool. Geol. J..

[25] Montalvo CI (2019). Chasicoan (late Miocene) vertebrate assemblage from Cerro Azul Formation, central Argentina. Geomorphological and biochronological considerations. J. South. Am. Earth Sci..

[26] Verzi DH, Montalvo CI (2008). The oldest South American Cricetidae (Rodentia) and Mustelidae (Carnivora): Late Miocene faunal turnover in central Argentina and the Great American Biotic Interchange. Palaeogeogr. Palaeocl Palaeoecol..

[27] Tomassini RL, Montalvo CI, Deschamps CM, Manera T (2013). Biostratigraphy and biochronology of the Monte Hermoso Formation (early Pliocene) at its type locality, Buenos Aires Province, Argentina. J. S Am. Earth Sci..

[28] MacFadden BJ (2005). Diet and habitat of toxodont megaherbivores (Mammalia, Notoungulata) from the late Quaternary of South and Central America. Quat. Res..

[29] MacFadden BJ, Shockey BJ (1997). Ancient feeding ecology and niche differentiation of Pleistocene mammalian herbivores from Tarija, Bolivia: morphological and isotopic evidence. Paleobiology.

[30] Sánchez B, Prado JL, Alberdi MT (2004). Feeding ecology, dispersal, and extinction of South American Pleistocene gomphotheres (Gomphotheriidae, Proboscidea). Paleobiology.

[31] González-Guarda E (2018). Multiproxy evidence for leaf-browsing and closed habitats in extinct proboscideans (Mammalia, Proboscidea) from Central Chile. Proc. Natl Acad. Sci. USA.

[32] Feranec RS, MacFadden BJ (2000). Evolution of the grazing niche in Pleistocene mammals from Florida: evidence from stable isotopes. Palaeogeogr. Palaeocl Palaeoecol..

[33] DeSantis, L. R. G., Feranec, R. S., MacFaddenm B. J. Effects of global warming on ancient mammalian communities and their environments. *PLoS ONE* 4: e5750. 10.1371/journal.pone.0005750 PMC2684586 19492043

[34] Vrba ES (1992). Mammals as key to evolutionary theory. J. Mammal..

[35] Moreno Bofarull A, Arias Royo A, Hernández Fernández M, Ortiz-Jaureguizar E, Morales J (2008). Influence of continental history on the ecological specialization and macroevolutionary processes in the mammalian assemblage of South America: Differences between small and large mammals. BMC Evol. Biol..

[36] Stock, B. C., Semmens, B. X. MixSIAR GUI user manual, v. 1.0 http://conserver.iugo-cafe.org/user/brice.semmens/MixSIAR [accessed April 2018] (2013).

[37] Cotte, M., Prevosti, F. J., Vizcaíno, S. F., Bocherens, H. Pleistocene trophic systems in the Pampean region (Buenos Aires province, Argentina): insights from C and O stable isotopes. *SVP 74th Annual Meeting*. Abstract volume **113** (2014).

[38] Grimes ST, Mattey DP, Hooker JJ, Collinson ME (2003). Paleogene paleoclimate reconstruction using oxygen isotopes from land and freshwater organisms: the use of multiple paleoproxies. Geochim. Cosmochim. Ac.

[39] Verzi DH, Deschamps CM, Montalvo CI (2008). Biostratigraphy and biochronology of the Late Miocene of central Argentina: evidence from rodents and taphonomy. Geobios.

[40] Prevosti FJ, Forasiepi AM, Zimicz N (2013). The evolution of the Cenozoic terrestrial mammalian predator guild in South America: competition or replacement?. J. Mammal. Evol..

[41] Haug GH, Tiedemann R, Zahn R, Ravelo AC (2001). Role of Panama uplift on oceanic freshwater balance. Geol..

[42] Zachos JC, Dickens GR, Zeebe RE (2008). An early Cenozoic perspective on greenhouse warming and carbon-cycle dynamics. Nat..

[43] Webb SD (1976). Mammalian faunal dynamics of the Great American Biotic Interchange. Paleobiology.

[44] Tonni EP, Alberdi MA, Prado JL, Bargo MS, Cione AL (1992). Changes of mammal assemblages in the Pampean region (Argentina) and their relation with the Plio-Pleistocene boundary. Palaeogeogr. Palaeocl Palaeoecol..

[45] Prevosti, F. J., Forasiepi, A. M. *Evolution of South American mammalian predators during the Cenozoic: paleobiogeographic and paleoenvironmental contigencies*. Springer Geology. 196 (2018).

[46] Domingo L (2013). Late Neogene and Early Quaternary paleoenvironmental and paleoclimatic conditions in Southwestern Europe: isotopic analyses on mammalian taxa. PLOS ONE.

[47] R Core Team. R: a language and environment for statistical computing. v. 3.1.1. *R Foundation for Statistical Computing*, Vienna, Austria, http://www.R-project.org.

[48] Jackson AL, Inger R, Parnell AC, Bearhop S (2011). Comparing isotopic niche widths among and within communities: SIBER – Stable Isotope Bayesian Ellipses in R. J. Anim. Ecol..

