# Supplementary material for: The Great American Biotic Interchange revisited: a new perspective from the stable isotope record of Argentine Pampas fossil mammals
Source: Sci Rep. 2020 Jan 31;10:1608. doi: 10.1038/s41598-020-58575-6 (PMC6994648; doi:10.1038/s41598-020-58575-6)
Supplement: Supplementary file 1 — Supplementary Information. [file 41598_2020_58575_MOESM1_ESM.pdf]

Supplementary Information for

The Great American Biotic Interchange revisited: a new perspective from the stable isotope record of Argentine Pampas fossil mammals

Laura Domingo[1, 2*], Rodrigo L. Tomassini[3], Claudia I. Montalvo[4], Dánae Sanz-Pérez[1], María Teresa Alberdi[5]

[1]Departamento de Geodinámica, Estratigrafía y Paleontología, Facultad Ciencias Geológicas, Universidad Complutense de Madrid, Madrid 28040, Spain. (ldomingo@ucm.es*, dasanz01@ucm.es) *Corresponding author.

[2]Earth and Planetary Sciences Department, University of California Santa Cruz, Santa Cruz, California 95064, U.S.A.

[3]INGEOSUR, Universidad Nacional del Sur (UNS)-CONICET, Bahía Blanca 8000, Argentina. (rodrigo.tomassini@yahoo.com.ar)

[4]Facultad de Ciencias Exactas y Naturales, Universidad Nacional de La Pampa, Santa Rosa 6300, Argentina. (cmontalvo@exactas.unlpam.edu.ar)

[5]Departamento de Paleobiología. Museo Nacional de Ciencias Naturales-CSIC, Madrid 28006, Spain. (alberdi.maite@gmail.com)

This PDF file includes:

Supplementary Text
Supplementary Fig. S1
Captions for Supplementary Tables S1 to S6
References for Supplementary Text citations

Other supplementary materials for this manuscript include the following:

Tables S1 to S6

Supplementary Text

Significance of stable isotope analyses on mammalian fauna. Measurement of $\delta^{13}C$ values of bioapatite allows the characterization of the diet of extinct taxa, as well as the reconstruction of past habitat preferences. For herbivorous mammals, the $\delta^{13}C$ value is related to the $\delta^{13}C$ value of the ingested vegetation, which varies depending on plant photosynthetic pathways; C_3, C_4, CAM (Crassulacean Acid Metabolism), as well as ecological factors (e.g., aridity, canopy density) that affect fractionation during photosynthesis[1, 2]. The $\delta^{13}C$ values of carnivores reflect the isotopic values of ingested prey[3], but trophic fractionation must be accounted for before predator-prey inferences are undertaken. Clementz *et al.*[4] observed ^{13}C-depleted values of ~1.3‰ (range: 1.1–1.5‰) in tooth enamel of North American wolves when compared to ungulate prey. Following this premise, carnivore $\delta^{13}C$ value can be used as a complement to herbivore carbon isotope patterns to track environmental modification over a considered time span.

The $\delta^{18}O$ values of mammalian bioapatite record the $\delta^{18}O$ value of body water (offset by a temperature-dependent fractionation); the $\delta^{18}O$ value of body water, in turn, reflects the fluxes and $\delta^{18}O$ value of oxygen entering and exiting the body[5]. Within a taxon, variations in body water $\delta^{18}O$ values can be interpreted as chiefly reflecting changes in the isotopic composition of ingested water, which varies with mean annual temperature and aridity[6].

Establishment of vegetation $\delta^{13}C$ cut-off values. The $\delta^{13}C$ values of herbivore tooth enamel bioapatite track the $\delta^{13}C$ values of the plants they consume, offset by a fractionation factor associated with carbonate equilibria and metabolic processes[7, 8]. In order to set vegetation $\delta^{13}C$ cut-off values in our study, we have taken into account the

study by Tejada-Lara et al.[9] who warned about the relationship between dietary-bioapatite $\delta^{13}C$ enrichment ($\varepsilon^*_{diet-enamel}$) and body mass. Until a more in-depth study relating $\varepsilon^*_{diet-enamel}$ and body mass of the selected taxa from our study is performed, we have established conservative vegetation $\delta^{13}C$ cut-off ranges by considering three different $\varepsilon^*_{diet-enamel}$ values:

- $\varepsilon^*_{diet-enamel}$ = +14.1‰[7] for notoungulates, litopterns and ungulates, as well as for carnivoran $\delta^{13}C$ values corrected for predator-prey trophic offset. By using this $\varepsilon^*_{diet-enamel}$ value, the $\delta^{13}C$ threshold between C_3-dominated diet and intermediate C_3-C_4 diet is set at ~ -9‰ to -8‰, whereas the $\delta^{13}C$ threshold between intermediate C_3-C_4 diet and C_4-dominated diet is at ~ -2‰ to -1‰ (Fig. 2B, C, F, G, H, I, J).
- $\varepsilon^*_{diet-enamel}$ = +12.8‰ for rodents. This value is the one calculated for the European rabbit *Oryctolagus cuniculus*[8], whose body size ranges between 1.5 to 2.5 kg. To the best of our knowledge, there are no studies focused on the estimation of dietary-bioapatite $\delta^{13}C$ enrichment in modern rodents of large body masses such as those endemic from South American. By using a $\varepsilon^*_{diet-enamel}$ of +12.8‰, the $\delta^{13}C$ threshold between C_3-dominated diet and intermediate C_3-C_4 diet is set at ~ -10‰ to -9‰, whereas the $\delta^{13}C$ threshold between intermediate C_3-C_4 diet and C_4-dominated diet is situated at -3‰ to -2‰ (Fig. 2A).
- $\varepsilon^*_{diet-enamel}$ = +15.6‰ for xenarthrans based on the estimated dietary-bioapatite $\delta^{13}C$ enrichment for the extinct ground sloth *Mylodon darwinii* by Tejada-Lara et al.[9]. By using a $\varepsilon^*_{diet-enamel}$ of +15.6‰, the $\delta^{13}C$ threshold between C_3-dominated diet and intermediate C_3-C_4 diet is set at ~ -7‰ to -6‰, and $\delta^{13}C$ threshold between intermediate C_3-C_4 diet and C_4-dominated diet is set at 0‰ to 1‰ (Fig. 2D, E). For our study, we consider that a $\varepsilon^*_{diet-enamel}$ of +15.6‰ is a very conservative value and it may not be applicable to all the

xenarthrans due to their different body masses. In the case of cingulates, $\varepsilon^*_{diet-enamel}$ values have not been estimated yet for fossil taxa, so no definitive conclusions can be drawn as far as vegetation $\delta^{13}C$ cut-off values are concerned.

Materials and methods. Analysed teeth belong to the paleontological collections of the Facultad de Ciencias Exactas y Naturales de la Universidad Nacional de La Pampa (GHUNLPam; Santa Rosa, La Pampa), Universidad Nacional del Sur (PV-UNS; Bahía Blanca, Buenos Aires), Universidad Nacional del Centro de la Provincia de Buenos Aires (FCS; Olavarría, Buenos Aires), Universidad Nacional de San Luis (MHIN-UNSL-GEO; San Luis, San Luis), Museo Municipal de Ciencias Naturales Carlos Darwin (MD-PDB and MD-FM; Punta Alta, Buenos Aires), Museo Municipal de Ciencias Naturales Vicente Di Martino (MMH-CH and MMH-FM; Monte Hermoso, Buenos Aires), Museo de La Plata (MLP; La Plata, Buenos Aires), and Museo Argentino de Ciencias Naturales Bernardino Rivadavia (MACN; Buenos Aires).

The chronology of Late Cenozoic vertebrate localities in the Argentinian Pampean region was historically limited by the shortage of absolute numerical ages. Nevertheless, increasing paleontological research in some localities over the last decades, with accurate stratigraphic and geographical control, along with the application of multidisciplinary studies (e.g., sedimentology, taphonomy, sequence stratigraphy, magnetostratigraphy) have led to the development of a complete biostratigraphic layout for this time interval including the definition of several stages/ages (10, 11, and references therein). In this context, only two of the selected localities in this study show absolute ages for its fossil-bearing levels: Arroyo Chasicó (9.23 ± 0.09 Ma, Late Miocene; see 12) and Playa del Barco (16,440 ±

320 years[13] or 19,849 cal years BP[14]). Therefore, the chronology proposed in our study for the faunal assemblages from the selected localities is mainly based on the biostratigraphic and biochronological data proposed by different authors, including the stages/ages Chasicoan (Late Miocene), Huayquerian (Late Miocene), Montehermosan (Early Pliocene), Ensenadan (Early Pleistocene) and Lujanian (Late Pliestocene). The units defined for the Neogene associations are based on octodontoid rodents (see 11, 15–21) that, on account of its small tooth size, were not included in the present study.

We analysed carbon and oxygen isotope composition on the carbonate fraction of 338 mammalian bioapatite (tooth enamel and orthodentine) samples spanning a temporal interval from between ~9.5 million years (Late Miocene, Chasicoan) to ~12,000 years (Late Pleistocene, Lujanian) of the Argentine Pampas (La Pampa and Buenos Aires provinces) (Fig. 1, Supplementary Tables S1 and S2). Additionally, 72 bioapatite samples belonging to Pleistocene localities from the Buenos Aires province were included in the database from a previous study[22] (Supplementary Tables S1 and S2). All the sampled taxa have body masses > 1 kg.

Sample chemical treatment followed the one described in Domingo et al.[23] for carbonate in bioapatite. In order to remove any trace of water within the treated bioapatite powder samples that may affect $\delta^{18}O$ values, they were roasted at 65ºC under vacuum for one hour and a half before IRMS analysis. Analyses were conducted at the Stable Isotope Laboratory of the University of California Santa Cruz using a ThermoScientific MAT253 dual inlet isotope ratio mass spectrometer coupled to a ThermoScientific Kiel IV carbonate device. Temperature of reaction with H_3PO_4 was 75ºC. The standards used were Carrara Marble (CM; $\delta^{13}C$ = 2.05‰ and $\delta^{18}O$ = -1.91‰), NBS-18 ($\delta^{13}C$ = -5.03‰ and $\delta^{18}O$ = -

23.01‰) and in-house coral aragonite Atlantis II ($\delta^{13}C$ = 0.87‰ and $\delta^{18}O$ = 3.41‰). The standard deviations for repeated measurements of CM (n = 53), NBS-18 (n = 24) and Atlantis II (n = 25) were 0.04‰, 0.04‰ and 0.09‰ for $\delta^{13}C$, respectively, and 0.06‰, 0.09‰ and 0.07‰ for $\delta^{18}O$, respectively. Duplicate analyses were carried out for ~10% of the samples (n = 35). The average absolute differences for $\delta^{13}C$ and $\delta^{18}O$ values were 0.02‰ and 0.14‰, respectively, and the standard deviations of these average differences were 0.06‰ and 0.11‰ for $\delta^{13}C$ and $\delta^{18}O$ values, respectively.

We analysed oxygen isotope composition on the phosphate fraction of 322 tooth enamel samples: 271 samples were analysed for the first time in this study and 51 samples were taken from Domingo et al.[22] (Supplementary Table S2). Sample chemical treatment followed the one described in Domingo et al.[23] for phosphate in bioapatite. $\delta^{18}O_{PO4}$ values were measured at the Stable Isotope Laboratory of the University of California Santa Cruz using a Thermo Finnigan Delta plus XP isotope ratio mass spectrometer coupled via continuous flow to a high temperature conversion elemental analyzer (TCEA). Repeated measurements of in-house and international standards are precise to within 0.3‰. The standards used were in-house Ag_3PO_4 Fisher standard ($\delta^{18}O$=8.4‰), IAEA601 ($\delta^{18}O$=23.2‰) and IAEA602 ($\delta^{18}O$=71.4‰). The $\delta^{18}O$ standard deviations for repeated measurements of Fisher standard (n = 30), IAEA601 (n = 17) and IAEA602 (n = 17) were 0.4‰, 0.3‰ and 0.7‰. Replicate $\delta^{18}O_{PO4}$ analyses were carried out in ~30% (n = 90) of the selected samples. The average absolute difference for $\delta^{18}O_{PO4}$ was 0.03‰ and the standard deviation of this average difference was 0.06‰.

We used R software version 3.1.1. (http://www.r-project.org/)[24] for statistical analysis of the isotopic results. Shapiro-Wilk tests and Q-Q plots revealed normal distributions for

isotopic data and therefore, we performed parametric analyses to test for differences in the mean of isotopic values among fossil localities (Supplementary Table S3) and taxa (Supplementary Table S6). Significance level was set at $p = 0.05$.

Isotopic signatures yield information about both the resources used by an organism and the habitat in which it lives. The term "isotopic niche" refers to an area (δ-space) with isotope values (δ-values) as coordinates, so variance in δ-space among individuals is related to realized niche width[25]. We evaluated isotopic niches for Pampean fossil mammals using SIBER (Stable Isotope Bayesian Ellipses in R[26]), which is a subset of functions within the mixing model SIAR package. SIBER is a multivariate ellipse-based metrics developed as an alternative to previous convex hull methods. It allows solving for sample size bias inherent to previous methods by using a Bayesian approach[26]. To assess isotopic niche by means of SIBER it is necessary to use at least two isotopic systems (in our case, $\delta^{13}C$ and $\delta^{18}O$), which permit to delimitate a 2D δ-space. SIBER also needs to be fed with at least three data point per taxon to retrieve ellipses. SIBER uses a Markov Chain Monte Carlo (MCMC) model-fitting algorithm to construct a standard ellipse area (SEA) that best fits each set of $\delta^{13}C$ and $\delta^{18}O$ from different members within a (paleo)community (i.e., different taxa). We evaluated the isotopic niche in those localities with a good representation of taxa (three or more orders) and where each order has a good sampling record to retrieve ellipses (three or more samples per order).

We used MixSIAR mixing model[27] to estimate the proportions of source (prey) contributions to a consumer (predator). This methodology was applied on those localities where there is carnivorous representation including the Late Miocene sites of Arroyo Chasicó, Quehué, Salinas Grandes de Hidalgo, and the Late Pleistocene sites of Playa del

Barco and Santa Rosa (Supplementary Table S5). Mixing models find the combinations of prey proportions that are mathematically feasible solutions that would explain consumer isotope values. With the aim of not compromising the discriminatory power of the mixing model due to a large number of sources, we kept the source number below 6. MixSIAR results (posterior probabilities of dietary proportions) are reported as the median and 95% Bayesian credible intervals of the likely contribution of each prey taxon to the tissue composition of the predators. MixSIAR uses a Markov Chain Monte Carlo (MCMC) model-fitting algorithm. We considered model results as satisfactory when they converged in probability space, as indicated by trace plots and Gelman-Rubin and Geweke tests provided in MixSIAR[27].

Diagenesis evaluation. In order to reconstruct past ecological and environmental conditions based on stable isotope information derived from bioapatite tissues, the diagenetic effect on the stable isotope imprint must be assessed. Several controls allowed us to consider that the original isotopic signal of Argentine fossil mammalian bioapatite is preserved and therefore, usable for reconstruction of past features.

Although oxygen in the phosphate fraction of bioapatite is regarded as most reliable than oxygen in the carbonate fraction, due to more stable P-O bonds in contrast to C-O bonds, it is possible to evaluate whether $\delta^{18}O$ values of the carbonate ($\delta^{18}O_{CO3}$) preserve a pristine signal by comparing these values to those shown by $\delta^{18}O$ values of the phosphate ($\delta^{18}O_{PO4}$). The rationale of this reasoning is based on the comparison of modern living mammalian $\delta^{18}O_{CO3}$ and $\delta^{18}O_{PO4}$ values. Bryant et al.[28] and Iacumin et al.[29] observed that if CO_3^{-2} and PO_4^{-3} in bioapatite are cogenetic equilibrium precipitates from body water at

relatively invariant mammalian body temperatures, there should be a consistent difference in $\delta^{18}O_{CO3}$ and $\delta^{18}O_{PO4}$ values (expressed as $\Delta^{18}O_{CO3-PO4} = \delta^{18}O_{CO3} - \delta^{18}O_{PO4}$). These authors observed a consistent range of values for this difference between ~ 8.6 to 9.1‰. Obtaining a $\Delta^{18}O_{CO3-PO4}$ value near this range for fossil mammalian bioapatite has been regarded as an indication that both phases retain pristine isotopic values. Supplementary Table S2 shows raw isotopic data for fossil mammalian taxa considered in this study. Mean $\Delta^{18}O_{CO3-PO4}$ value for the whole dataset is 8.9±0.7‰ (Supplementary Tables S1 and S2). These results point to a good preservation of the oxygen isotope composition of both, the carbonate and phosphate fractions of the sampled tooth enamel herein. As far as $\delta^{13}C$ tooth enamel values are concerned, there are no conclusive tests that categorically confirm its preservation. However, the fact that species cluster in bivariate isotope space, and that the relative positions of these clusters are consistent for some taxa, suggest that animal paleobiology, and not diagenesis, is the main driver of isotopic variation.

Xenarthrans´ isotopic signal needs to be regarded with extra caution due to their lack of enamel, implying the use of orthodentine as the analyzed bioapatite tissue. To address this issue, we conducted three different tests on each fossil locality: i) we checked whether the difference between $\delta^{18}O_{CO3}$ and $\delta^{18}O_{PO4}$ ($\Delta^{18}O_{CO3-PO4}$) is in the range for modern unaltered apatite (~8.6–9.1‰[28, 29]); ii) whether there was a high correlation between $\delta^{18}O_{CO3}$ and $\delta^{18}O_{PO4}$, when combining xenarthran values with isotopic results from the rest of contemporaneous fauna belonging to the same locality; and iii) whether there was a poor correlation between $\delta^{13}C$ and $\Delta^{18}O_{CO3-PO4}$ values, when combining xenarthran values and isotopic results provided by the rest of contemporaneous fauna belonging to the same locality. Supplementary Table S4 shows the results of these tests and they suggest that the

selected xenarthran orthodentine record is well preserved and therefore can be used to address the research questions exposed in the framework of this study. Furthermore, Figure 2 shows that xenarthran $\delta^{13}C$ values are within the $\delta^{13}C$ range shown by other taxa and, in the case of tardigrades, they also record a shift from the consumption of C_3 plants to a diet influenced by the ingestion of C_4 vegetation.

$\delta^{18}O$ change through the GABI temporal interval. Tooth enamel $\delta^{18}O$ data through time for the orders with the larger number of samples and that depict the most complete long-term sequences (Rodentia, Notoungulata and Litopterna) are shown in Figure S1. Obligate drinkers obtain most of their water from drinking, whereas non-obligate drinkers obtain water mainly from plant water and metabolic water[5]. Extinct rodent drinking behavior is difficult to assess due to their particular metabolic activity and ecological preferences (e.g., 30 and references therein). Some studies have considered that rodent tooth enamel $\delta^{18}O$ values ultimately reflect local water $\delta^{18}O$ values due to their limited dispersal ability[31]. The main pitfall lies in determining whether ancient rodents obtained most of its water through drinking or from food items. To the best of our knowledge there are no studies carried out on modern populations of Dinomyidae, Caviidae and Chinchillidae that may shed light on their water use; however, some paleoecological inferences were proposed for the extinct representatives of these families. Deschamps et al.[18, 32] indicated that the fossil capybaras (Caviidae) *Cardiatherium* were associated to fluvial/lacustrine/swampy paleoenvironments, similarly to modern counterparts (*Hydrochoerus hydrochaeris*), and therefore they might be good recorders of local hydrological conditions. Fields[33] suggested that fossil dinomyids occupied many different ecological niches, in some cases associated

to water bodies (see 34). Rasia[35] argued that fossil representatives of the chinchillid *Lagostomus* may have had similar ecophysiological traits to those observed in the extant species *L. maximus*, which occupies ecosystems with a highly variable water availability. It is also difficult to ascertain whether the endemic orders Notoungulata and Litopterna obtained most of their water through drinking or eating since they have no extant counterparts. In the case of notoungulates, the fact that our study includes different families (Hegetotheriidae, Mesotheriidae, Toxodontidae) with distinct body size, morphology, and lifestyle may point to differences in their water use and source[36, 37]. Mesotherids have been proposed to have a similar lifestyle to that observed in modern capybaras[38]. In the case of toxodonts, the plasticity observed in their feeding[39] may have conditioned their water use. Therefore, those feeding on C_3 to C_3-C_4 woodlands and grasslands, such as those from the Pampean region, may have drunk frequently to cover their body water requirements. In the case of litopterns, i) their more consistent body plan and dental features among the studied families (Macraucheniidae and Proterotheriidae), ii) the fact that machauchenids have been suggested to be cursorial species, and iii) closer affinities with extant perissodactyls[40] would suggest they may have been obligate drinkers. Fitted loess curves depicted in Supplementary Figure S1 show different patterns for each order probably reflecting particular water use, rather than recording global climatic trends. As mentioned in the case of the $\delta^{13}C$ record, there are no major biases driven by the specific families in each locality as there is a good representation of different families per fossil site. In the case of rodents, it can be observed an increase in $\delta^{18}O$ values since the Chasicoan (Late Miocene; Cerro La Bota and Arroyo Chasicó localities) with a peak in the Salinas Grandes de Hidalgo locality (Late Miocene, Huayquerian), recording an aridity

maximum, and a progressive decrease since this moment on, including Bajo Giuliani (Late Miocene, Huayquerian), Caleufú (Late Miocene/Early Pliocene; late Huayquerian) and Farola Monte Hermoso (Early Pliocene, Montehermosan) localities (Supplementary Figure S1A). This partially agrees with the studies carried out by Verzi *et al.*[16, 41], who based on an investigation of changes observed in rodent dental features, including an increase in molar hypsodonty, proposed a progressive rise in aridity across the Late Miocene. Notoungulate´s $\delta^{18}O$ curve also shows a rise followed by a drop, although the timing of this pattern is different from that recorded by rodents: the maximum $\delta^{18}O$ value is detected at the Farola Monte Hermoso locality (Early Pliocene, Montehermosan) (Supplementary Figure S1B). This would point to an increase in aridity and/or temperature since the Late Miocene to the Early Pliocene in the Pampean area; probably followed by a shift towards more humid and/or colder conditions in response to the progressive global cooling[42, 43]. The difference between rodent and notoungulate $\delta^{18}O$ curve may be due to body size contraints that determine geographical distribution and dispersion capacity. Furthermore, litoptern $\delta^{18}O$ curve shows a different trend to those shown by rodents and notoungulates, with a gradual decrease since the Late Miocene to the Pleistocene (Supplementary Figure S1C). This result may indicate that litopterns would have been better recorders of the gradual global cooling that took place globally since the Middle-Late Miocene to the Late Pleistocene[42, 43]. In the light of rodent, notoungulate and litoptern $\delta^{18}O$ records, values recorded by each of these orders may be driven by local, regional and/or global hydrological conditions, respectively, that might be closely related to their particular water use, ecologies and habitat preferences. Observed particular $\delta^{18}O$ inter-order differences allow us to consider this isotopic system in the assessment of habitat use, which along with

the evaluation of resource use can help us depict niche occupation and width by means of SIBER modeling (see main text).

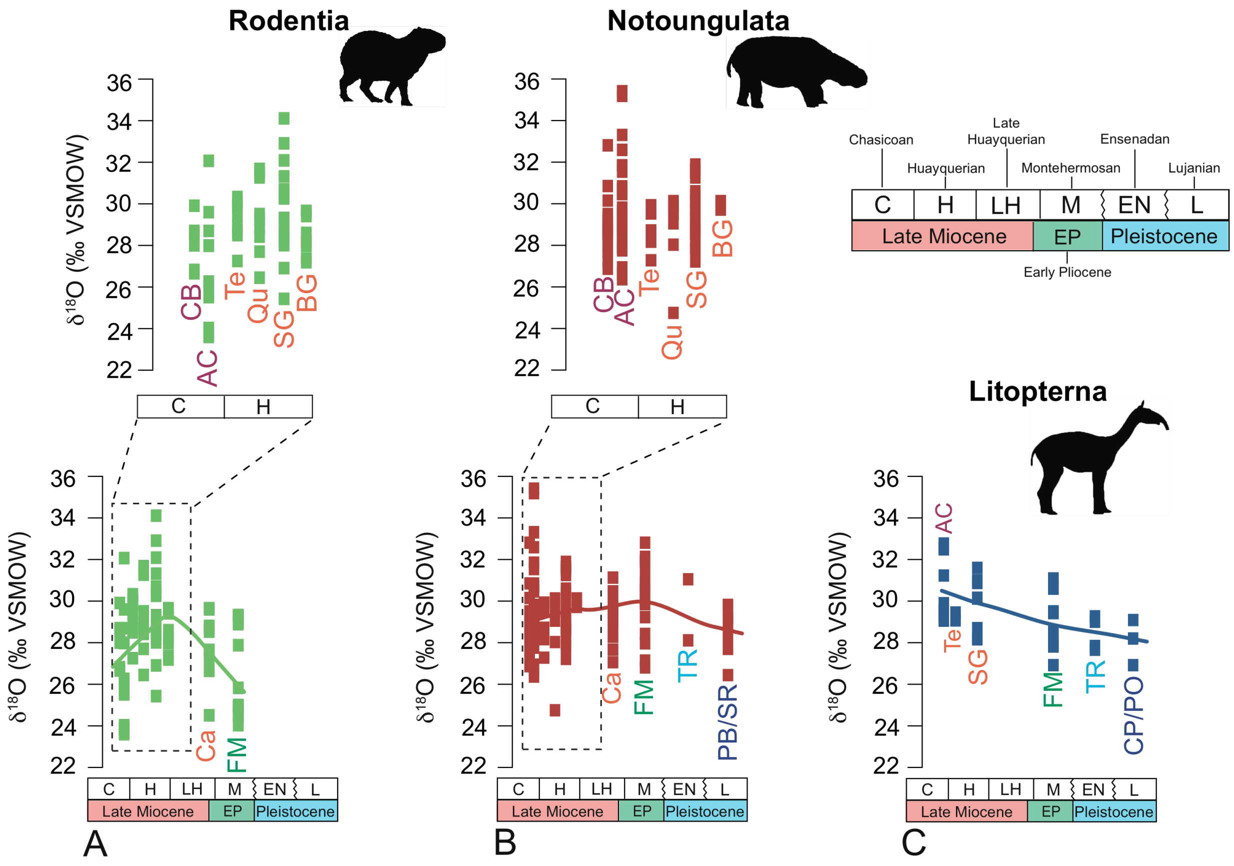

Figure S1. δ18O (‰, VSMOW) values and fitted loess curves of mammals from La Pampa and Buenos Aires provinces between the Late Miocene (Chasicoan) and the Late Pleistocene (Lujanian). A) Rodentia, B) Notoungulata and C) Litopterna. CB: Cerro La Bota, AC: Arroyo Chasicó, Te: Telén, Qu: Quehué, SG: Salinas Grandes de Hidalgo, BG: Bajo Giuliani, Ca: Caleufú, FM: Farola Monte Hermoso, TR: Toscas del Río de La Plata, PO: Paso Otero, CP: Cascada del Paleolama, PB: Playa del Barco, SR: Santa Rosa. Zig-zag lines in the chronology tables denote non-represented ages: the Montehermosan is followed by the Chapadmalalan, the Marplatan, the Ensenadan, the Bonaerian, and the Lujanian.

Supplementary Table captions

Table S1. Summary of stable isotope total dataset from all the Pampean localities analyzed in this study. Locality, province, epoch, Stage/Age, taxa, number of carbonate samples (#C), mean and standard deviation (SD) $\delta^{13}C$ (‰ VPDB), $\delta^{18}O_{CO3}$ (‰ VSMOW), number of phosphate samples (#P), mean and standard deviation (SD) $\delta^{18}O_{PO4}$ (‰ VSMOW) and difference between $\delta^{18}O_{CO3}$ and $\delta^{18}O_{PO4}$ values ($\Delta\ \delta^{18}O_{CO3}$-$\delta^{18}O_{PO4}$).

Table S2. Stable isotope total dataset from all the localities analyzed in this study. Locality, province, epoch, stage/age, collection number, order, family, genus/species, analysed tissue, $\delta^{13}C$ (‰ VPDB), $\delta^{18}O_{CO3}$ (‰ VPDB), $\delta^{18}O_{CO3}$ (‰ VSMOW), $\delta^{18}O_{PO4}$ (‰ VSMOW), difference between $\delta^{18}O_{CO3}$ and $\delta^{18}O_{PO4}$ values ($\Delta\ \delta^{18}O_{CO3}$-$\delta^{18}O_{PO4}$), and references.

Table S3. ANOVA and Tukey post-hoc tests for $\delta^{13}C$ (‰ VPDB), and $\delta^{18}O_{CO3}$ (‰ VSMOW) values of Pampean rodents, notoungulates and litopterns.

Table S4. Diagenetic tests for xenarthrans´ orthodentine. A) All taxa from different fossil sites, Stage/Age, $\Delta^{18}O_{CO3\text{-}PO4}$, Pearson´s r $\delta^{18}O_{CO3}$-$\delta^{18}O_{PO4}$, Pearson´s r $\delta^{13}C$-$\Delta^{18}O_{CO3\text{-}PO4}$ and B) only xenarthrans from different fossil sites, Stage/Age, Cingulata $\Delta^{18}O_{CO3\text{-}PO4}$, and Pilosa $\Delta^{18}O_{CO3\text{-}PO4}$.

Table S5. Predicted dietary proportions of predators from Arroyo Chasicó, Quehué, Salinas Grandes de Hidalgo, Playa del Barco, Santa Rosa derived from MixSIAR mixing model on $\delta^{13}C$ values of carnivores and herbivores.

Table S6. ANOVA and Tukey post-hoc tests for $\delta^{13}C$ (‰ VPDB), and $\delta^{18}O_{CO3}$ (‰ VSMOW) values among mammalian orders from those sites where SIBER was applied.

References Supplementary Information

1. Ehleringer, J. R., Monson, R. K. Evolutionary and ecological aspects of photosynthetic pathway variation. *Annu Rev Ecol Syst* **24**, 411–439 (1993).
2. Hayes, J. M. Fractionation of carbon and hydrogen isotopes in biosynthetic processes. *Rev Mineral Geochem* **43**, 225–277 (2001).
3. Domingo, L., Domingo, M. S., Koch, P. L., Morales, J., Alberdi, M. T. Carnivoran resource and habitat use in the context of a Late Miocene faunal turnover episode. *Palaeontology* **60**, 461–483 (2017).
4. Clementz, M. T., Fox-Dobbs, K., Wheatley, P. V., Koch, P. L., Doak, D.F. Revisiting old bones: coupled carbon isotope analysis of bioapatite and collagen as an ecological and palaeoecological tool. *Geol J* **44**, 605–620 (2009).
5. Kohn, M. J. Predicting animal $\delta^{18}O$: accounting for diet and physiological adaptation. *Geochim Cosmochim Ac* **60**, 4811–4829 (1996).
6. Luz, B., Cormie, A. B., Schwarcz, H. P. Oxygen isotope variations in phosphate of deer bones. *Geochim Cosmochim Ac* **54**, 1723–1728 (1990).
7. Cerling, T. E., Harris, J. M. Carbon isotope fractionation between diet and bioapatite in ungulate mammals and implications for ecological and paleoecological studies. *Oecologia* **120**, 347e363 (1999).
8. Passey, B. H. *et al.* Carbon isotope fractionation between diet, breath CO_2, and bioapatite in different mammals. *J Archaeol Sci* **32**, 1459–1470 (2005).
9. Tejada-Lara, J. V. *et al.* Body mass predicts isotope enrichment in herbivorous mammals. *Proc. R. Soc. B* **285**, 20181020 (2018).

10. Cione, A. L., Gasparini, G. M., Soibelzon, E., Soibelzon, L. H., Tonni, E. P. *The Great American Biotic Interchange. A South American perspective*. Springer briefs in Earth Sciences, 97 pp (2015).

11. Deschamps, C. M., Tomassini, R. L. Late Cenozoic vertebrates from the southern Pampean Region: systematic and bio-chronostratigraphic update in *Palinología del Meso-Cenozoico de Argentina - Volumen en homenaje a Mirta Elena Quattrocchio* (eds. Martínez, M., Olivera, D.), *Publicación Electrónica de la Asociación Paleontológica Argentina* **16**, 202–225 (2016).

12. Zárate, M. A. *et al.* Geology and geochronology of type Chasicoan (late Miocene) mammal-bearing deposits of Buenos Aires (Argentina). *J S Am Earth Sci* **23**, 81–90 (2007).

13. Tomassini, R. L., Montalvo, C. I., Manera, T., Oliva, C. Estudio tafonómico de los mamíferos pleistocenos del yacimiento de Playa del Barco (Pehuen Co), provincia de Buenos Aires, Argentina. *Ameghiniana* **47**, 137–152 (2010).

14. Prado, J. L., Martínez-Maza, C., Alberdi, M. T. Megafauna extinction in South America: A new chronology for the Argentine Pampas. *Palaeogeogr Palaeocl Palaeoecol* **425**, 41–49 (2015).

15. Verzi, D. H. The dental evidence on the differentiation of the ctenomyine rodents (Caviomorpha, Octodontidae, Ctenomyinae). *Acta Theriologica* **44**, 263–282 (1999).

16. Verzi, D. H., Montalvo, C. I. The oldest South American Cricetidae (Rodentia) and Mustelidae (Carnivora): Late Miocene faunal turnover in central Argentina and the Great American Biotic Interchange. *Palaeogeogr Palaeocl Palaeoecol* **267**, 284–291 (2008).

17. Verzi, D. H., Deschamps, C. M, Montalvo, C. I. Biostratigraphy and biochronology of the Late Miocene of central Argentina: evidence from rodents and taphonomy. *Geobios* **41**, 145–155 (2008).

18. Tomassini, R. L., Montalvo, C. I., Deschamps, C. M., Manera, T. Biostratigraphy and biochronology of the Monte Hermoso Formation (early Pliocene) at its type locality, Buenos Aires Province, Argentina. *J S Am Earth Sci* **48**, 31–42 (2013).

19. Deschamps, C. M., Vucetich, M. G., Montalvo, C. I., Zárate, M. A. Capybaras (Rodentia, Hydrochoeridae, Hydrochoerinae) and their bearing in the calibration of the late Miocene-Pliocene sequences of South America. *Journal of South American Earth Sicences* **48**, 145–158 (2013).

20. Sostillo, R., Montalvo, C. I., Verzi, D. H. A new species of *Reigechimys* (Rodentia, Echimyidae) from the Late Miocene of Central Argentina and the evolutionary pattern of the lineage. *Ameghiniana* **51**, 284–294 (2014).

21. Montalvo, C.I. *et al.* Chasicoan (late Miocene) vertebrate assemblage from Cerro Azul Formation, central Argentina. Geomorphological and biochronological considerations. *Journal of South American Earth Sciences* **95**, 102218 (2019).

22. Domingo, L., Prado, J. L., Alberdi, M. T. The effect of paleoecology and paleobiogeography on stable isotopes of Quaternary mammals from South America. *Quaternary Sci Rev* **55**, 103–113 (2012).

23. Domingo, L. *et al.* Late Neogene and Early Quaternary paleoenvironmental and paleoclimatic conditions in Southwestern Europe: isotopic analyses on mammalian taxa. *PLOS ONE* **8**, e63739 (2013).

24. R Core Team. R: a language and environment for statistical computing. v. 3.1.1. *R Foundation for Statistical Computing*, Vienna, Austria. http://www.R-project.org. (2008)

25. Newsome, S. D., Yeakel, J. D., Wheatley, P. V., Tinker, M. T. Tools for quantifying isotopic niche space and dietary variation at the individual and population level. *J Mammal* **93**, 329–341 (2012).

26. Jackson, A. L., Inger, R., Parnell, A. C., Bearhop, S. Comparing isotopic niche widths among and within communities: SIBER – Stable Isotope Bayesian Ellipses in R. *J Anim Ecol* **80**, 595–602 (2011).

27. Stock, B. C., Semmens, B. X. MixSIAR GUI user manual, v. 1.0. http://conserver.iugo-cafe.org/user/brice.semmens/MixSIAR [accessed April 2018] (2013)

28. Bryant, J. D., Koch, P. L., Froelich, P. N., Showers, W. J., Genna, B. J. Oxygen isotope partitioning between phosphate and carbonate in mammalian apatite. *Geochim Cosmochim Ac* **60**, 5145–5148 (1996).

29. Iacumin, P., Bocherens, H., Mariotti, A., Longinelli, A. Oxygen isotope analyses of coexisting carbonate and phosphate in biogenic apatite: a way to monitor diagenetic alteration of bone phosphate?. *Earth Planet Sci Lett* **142**, 1–6 (1996).

30. Royer, A. *et al.* What does the oxygen isotope composition of rodent teeth record?. *Earth Planet Sc Lett* **361**, 258–271 (2013).

31. Héran, M. A., Lécuyer, C., Legendre, S. Cenozoic long-term terrestrial climatic evolution in Germany tracked by $\delta^{18}O$ of rodent tooth phosphate. *Palaeogeogr Palaeocl Palaeoecol* **285**, 331–342 (2010).

32. Deschamps, C. M., Olivares, A. I., Vieytes, E. C., Vucetich, M. G. The oldest capybaras (Rodentia, Hydrochoeridae; Late Miocene of Argentina): ontogeny and diversity. *J Vertebr Paleontol* **27**, 683–692 (2007).

33. Fields, R. W. Hystricomorph rodents from the late Miocene of Colombia, South America. *University of California Publications in Geological Sciences* **32**, 273-404 (1957).

34. Rinderknecht, A., Blanco, R. The largest fossil rodent. *Proc R Soc B-Biol Sci* **275**, 923–928 (2008).

35. Rasia, L. L. Los Chinchillidae (Rodentia, Caviomorpha) fósiles de la República Argentina: sistemática, historia evolutiva y biogeográfica, significado bioestratigráfico y paleoambiental. PhD Thesis, Facultad de Ciencias Naturales y Museo, Universidad Nacional de La Plata, La Plata, 381 pp (2016).

36. Billet, G. Phylogeny of the Notoungulata (Mammalia) based on cranial and dental characters. *J Syst Palaeontol* **9**, 481–497 (2011).

37. Croft, D. A. Horned armadillos and rafting monkeys in *Life of the past* (ed. Farlow, J. O.) 304 pp (Indiana University Press, 2017).

38. Bond, M., Cerdeño, E., López, G. Los ungulados nativos de América del Sur in *Evolución biológica y climática de la región pampeana durante los últimos cinco millones de años* (eds. Alberdi, M. T., Leone, G., Tonni, E. P.) 257–276 (CSIC Monografías 12, Madrid, 1995).

39. MacFadden, B. J. Diet and habitat of toxodont megaherbivores (Mammalia, Notoungulata) from the late Quaternary of South and Central America. *Quat. Res.* **64**, 113–124 (2005).

40. Westbury, M., *et al.* A mitogenomic timetree for Darwin's enigmatic South American mammal *Macrauchenia patachonica*. Nat. Comm. **8**, 15951 (2017).

41. Verzi, D. H., Vieytes, E. C., Montalvo, C. I. Dental evolution in *Neophanomys* (Rodentia, Octodontidae) from the late Miocene of central Argentina. *Geobios* **44**, 621–633 (2011).

42. Haug, G. H. *et al.* North Pacific seasonality and the glaciation of North America 2.7 million years ago. *Nature* **433**, 821–825 (2005).

43. Zachos, J. C., Dickens, G. R., Zeebe, R. E. An early Cenozoic perspective on greenhouse warming and carbon-cycle dynamics. *Nature* **451**, 280–283 (2008).
