# Supplementary figures and images for: The Great American Biotic Interchange revisited: a new perspective from the stable isotope record of Argentine Pampas fossil mammals
Source: Sci Rep. 2020 Jan 31;10:1608. doi: 10.1038/s41598-020-58575-6 (PMC6994648; doi:10.1038/s41598-020-58575-6)

## Rodentia

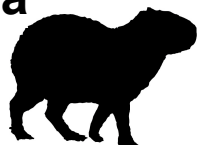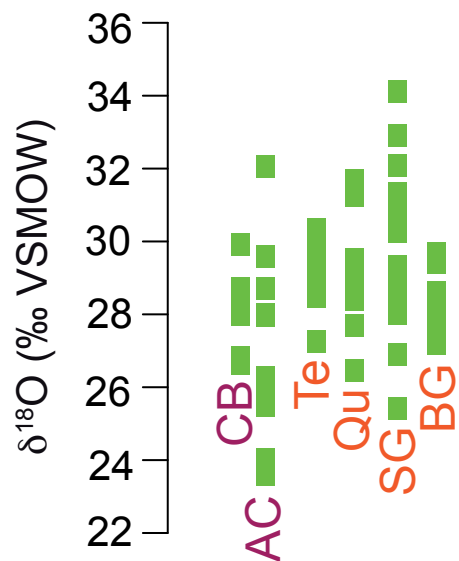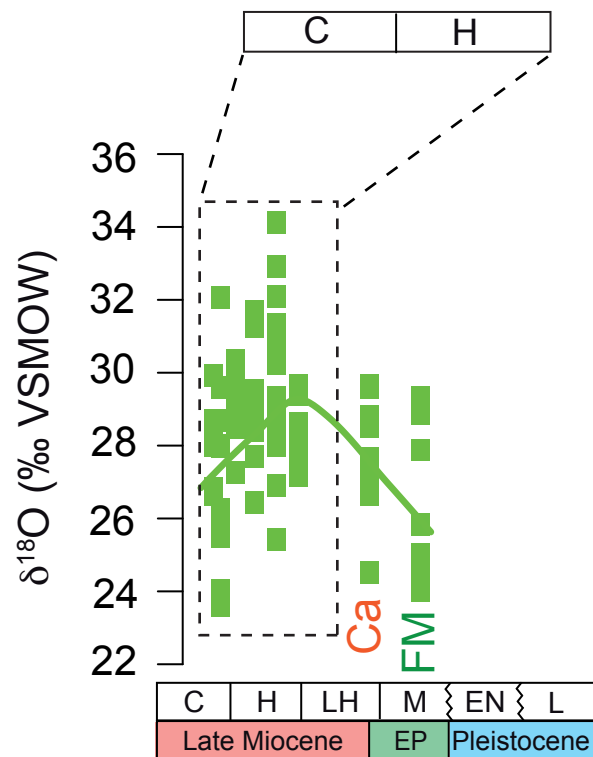

A

## Notoungulata

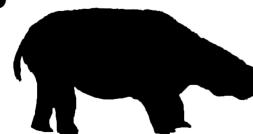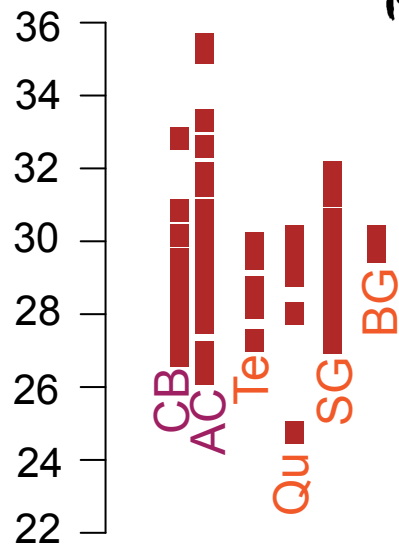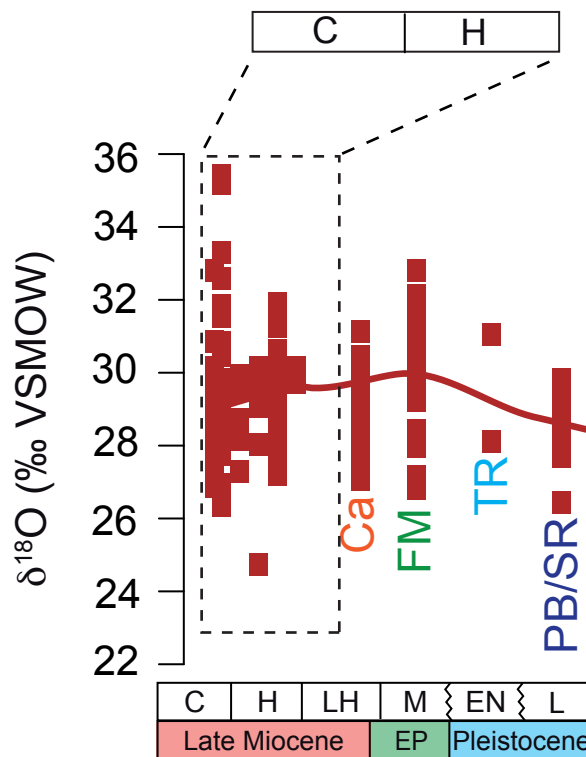

B

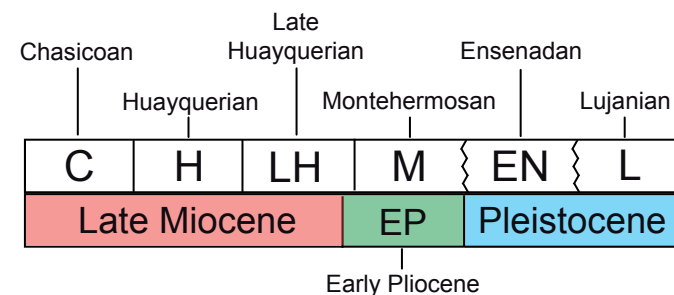

## Litopterna

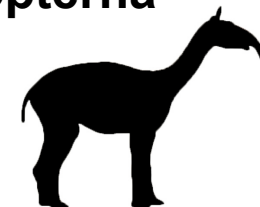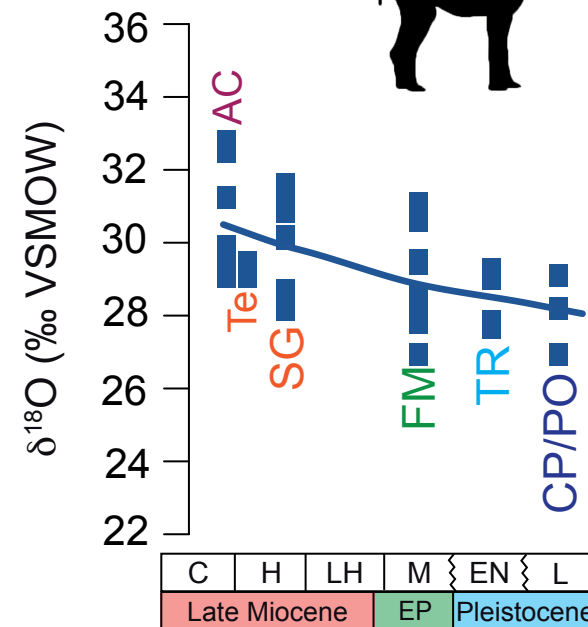

C

Supplement: Supplementary file 2 — Supplementary Information 2. [file 41598_2020_58575_MOESM2_ESM.pdf]
