# Supplementary material for: The Great American Biotic Interchange revisited: a new perspective from the stable isotope record of Argentine Pampas fossil mammals
Source: Sci Rep. 2020 Jan 31;10:1608. doi: 10.1038/s41598-020-58575-6 (PMC6994648; doi:10.1038/s41598-020-58575-6)
Supplement: Supplementary file 6 — Supplementary Information 6. [file 41598_2020_58575_MOESM6_ESM.doc]

| **A. ALL TAXA**  **Site** | **Stage/Age** | **Δ^18^O_CO3-PO4_**  **(‰ VSMOW)** | **Pearson´s r**  **δ^18^O_CO3_-δ^18^O_PO4_** | **Pearson´s r**  **δ^13^C- Δ^18^O_CO3-PO4_** |
| --- | --- | --- | --- | --- |
| Santa Rosa | Lujanian | 9.0±0.1 | 0.98 | 0.07 |
| Playa del Barco | Lujanian | 8.7±0.5 | 0.90 | 0.12 |
| Farola Monte Hermoso | Montehermosan | 9.2±0.5 | 0.98 | 0.07 |
| Caleufú | late Huayquerian | 9.0±0.2 | 0.99 | 0.07 |
| Salinas Grandes de Hidalgo | Huayquerian | 8.6±0.7 | 0.89 | 0.11 |
| Quehué | Huayquerian | 9.1±0.4 | 0.98 | 0.22 |
| Telén | Huayquerian | 9.0±0.3 | 0.95 | 0.20 |
| Arroyo Chasicó | Chasicoan | 8.9±0.2 | 0.99 | 0.23 |
| **B. XENARTHRA**    **Site** | **Stage/Age** | **CINGULATA**  **Δ^18^O_CO3-PO4_**  **(‰ VSMOW)** | **PILOSA**  **Δ^18^O_CO3-PO4_**  **(‰ VSMOW)** |  |
| Santa Rosa | Lujanian | - | 9.0±0.1 |  |
| Playa del Barco | Lujanian | - | 9.1±0.4 |  |
| Farola Monte Hermoso | Montehermosan | - | 9.1±0.4 |  |
| Caleufú | late Huayquerian | - | 9.1±0.2 |  |
| Salinas Grandes de Hidalgo | Huayquerian | 9.2 | 8.6 |  |
| Quehué | Huayquerian | 9.4±0.01 | - |  |
| Telén | Huayquerian | 8.9±0.2 | - |  |
| Arroyo Chasicó | Chasicoan | 9.1 | 9.0±0.01 |  |

**Table S4.** Diagenetic tests for xenarthrans´ orthodentine. A) All taxa from different fossil sites, Stage/Age, ∆^18^O_CO3-PO4_, Pearson´s r δ^18^O_CO3_-δ^18^O_PO4_, Pearson´s r δ^13^C-∆^18^O_CO3-PO4_ and B) only xenarthrans from different fossil sites, Stage/Age, Cingulata ∆^18^O_CO3-PO4_, and Pilosa ∆^18^O_CO3-PO4_.
